# EGFR-Targeted Immunotoxin Exerts Antitumor Effects on Esophageal Cancers by Increasing ROS Accumulation and Inducing Apoptosis via Inhibition of the Nrf2-Keap1 Pathway

**DOI:** 10.1155/2018/1090287

**Published:** 2018-11-25

**Authors:** Yun Yang, Ziyin Tian, Yanke Ding, Xiaojing Li, Ziheng Zhang, Liu Yang, Fangyu Zhao, Feng Ren, Rui Guo

**Affiliations:** ^1^School of Basic Medical Sciences, Xinxiang Medical University, Xinxiang, China; ^2^State Key Laboratory of Antibody Medicine and Targeted Therapy, Shanghai, China

## Abstract

Previously, we developed a novel EGFR-targeted antibody (denoted as Pan), which has superior antitumor activity against EGFR-overexpressed tumors. However, it shows marginal effect on the growth of esophageal cancers. Therefore, the variable region of Pan was fused to a fragment of Pseudomonas exotoxin A (PE38) to create the immunotoxin, denoted as Ptoxin (PT). Results indicated that PT shows more effective antitumor activity as compared with Pan both on EGFR-overexpressed KYSE-450 and KYSE-150 esophageal cancer cells, especially on KYSE-450 cells. Moreover, treatment of PT induces regression of KYSE-450 tumor xenografts in nude mice. Furthermore, we investigated the potential mechanism involved in the enhanced antitumor effects of PT. Data showed that PT was more potent in reducing the phosphorylation of EGFR and ERK1/2. More importantly, we for the first time found that PT was more effective than Pan in inducing ROS accumulation by suppression of the Nrf2-Keap1 antioxidant pathway, and then induced apoptosis in KYSE-450 esophageal cancer cells, which may partly explain the more sensitive response of KYSE-450 to PT treatment. To conclude, our study provides a promising therapeutic approach for immunotoxin-based esophageal cancer treatment.

## 1. Introduction

Esophageal cancer is one of the most serious tumor diseases worldwide. Particularly, the northern region in the Henan Province of China has the highest incidence of esophageal cancer, especially the esophageal squamous cell carcinoma (ESCC) [1]. Current treatment options for patients with esophageal cancer include surgery followed by radiation and chemotherapy; however, the prognosis of esophageal cancer is poor with a 5-year survival rate less than 10% [1, 2]. Therefore, more potent therapeutic approaches are urgently needed to improve survival of patients with esophageal cancer.

Epidermal growth factor receptor (EGFR), a transmembrane receptor kinase, is frequently overexpressed in various types of human cancers, including esophageal cancer [3, 4]. Several anti-EGFR monoclonal antibodies (mAbs) such as cetuximab and panitumumab are established agents in the treatment of colorectal and head and neck cancer [5]. However, EGFR-targeted antibodies have shown limited activity against esophageal cancer [3].

Immunotoxin, which is the therapeutic molecule consisting of an antibody fragment fused to a protein cytotoxin, has been developed for treating many malignant cancers [6, 7]. It combined the specific targeting of a tumor-expressed receptor with the potent cell killing of cytotoxic toxin [8]. PE38, a truncated version of Pseudomonas exotoxin A (PE), is one of the most widely applied toxins for the development of immunotoxins [9, 10]. It functions through delivering a cytotoxic effect in the cytosol and resulting in cell death [11]. As we know, researchers have increasingly focused on genetically engineered single-chain variable region antibody fragment (scFv) consisting of the heavy- and light-chain variable regions (VH and VL) fused to immunotoxins, which target the antigens specifically on tumor-expressed cells, such as EGFR and CD20 [12, 13]. Over the decades, many bacterial- or plant-based immunotoxins have been developed with the aim of targeting a variety of cancers reliant upon EGFR overexpression [8].

Recently, several studies revealed that EGFR or HER2 inhibitors may exhibit antitumor effects in association with persistent promotion of reactive oxygen species (ROS) generation and induced apoptosis [5, 14, 15]. Khalil et al. indicated that the HER2-targeted therapeutic treatment promoted reactive oxygen species (ROS), glutathione (GSH) depletion, reduction in nuclear factor erythroid 2-related factor 2 (Nrf2) levels, and inhibition of Nrf2 function in ovarian cancer cell lines [14]. Nrf2 was deemed as a redox-sensitive master regulator of a variety of crucial antioxidant genes. Under physiological conditions, its steady-state level is regulated by Kelch-like ECH-associated protein 1 regulator (Keap1) that could mediate Nrf2 degradation by the ubiquitin proteasome pathway [15–17]. Leone et al. demonstrated that vorinostat, in combination with EGFR inhibitor, synergistically induced proapoptotic effects by altering redox homeostasis via modulating the antioxidant c-Myc-Nrf2-Keap1 pathway [15].

Previously, we constructed and characterized a novel anti-EGFR mAb, Pan, which has shown effective antitumor activity [18, 19]. In the present study, we reported the novel immunotoxin PT by fusing the scFv of Pan to 38 kDa truncated fragment of PE and investigated its potent antitumor potency against esophageal cancer cells *in vitro* and *in vivo*. Results indicated that the EGFR-ERK1/2 pathway was inhibited in KYSE-450 and KYSE-150 cells upon PT treatment. More importantly, we for the first time found that PT may exert antitumor activity on KYSE-450 cells through inducing ROS production and apoptosis via inhibition of the Nrf2-Keap1 pathway.

## 2. Materials and Methods

### 2.1. Cell Lines and Animals

The human esophageal cancer cell lines KYSE-450, KYSE-150, KYSE-510, and EC9706 were purchased from the American Type Culture Collection (ATCC). Six-week-old female BALB/c nude mice were obtained from the Shanghai Experimental Animal Center of Chinese Academy of Sciences (Shanghai, China). All animals were treated in accordance with guidelines of the Committee on Animals of the Xinxiang Medical University.

### 2.2. Preparation of PT

As previously reported, the variable region of heavy chain and light chain (scFv) of Pan was cloned into the pET-28a(+) vector containing PE38KDEL fragment, respectively [20, 21]. Then, the construct was transformed into BL21 (DE3) cells and expressed in accordance with the corresponding protocol. Finally, the resulted immunotoxin with His-tag was purified through immobilized metal ion affinity chromatography (IMAC) using Ni-NTA purification resin and was verified by SDS-PAGE.

### 2.3. EGFR Binding Measurements by ELISA

The assay was performed as described previously [18, 19]. Briefly, 96-well plates (Nunc) were coated with recombinant EGFR-Fc (Sino Biological) and blocked with phosphate-buffered saline (PBS) containing 10% nonfat dried milk. Indicated concentrations of PT were added to the plates and incubated for 1 hour at room temperature. After a wash step, bound antibody was detected with horseradish peroxidase- (HRP-) conjugated anti-His-tag antibody for 30 min at 37°C followed by the addition of 3,3′,5,5-tetramethylbenzidine (TMB) substrate reagent. Absorbance was read at 450 nm. Data were fitted using a sigmoidal 4-parameter curve.

### 2.4. EGFR Expression Analysis

For cell surface EGFR expression analysis, esophageal cells were incubated on ice with anti-EGFR antibody (Cell Signaling Technology) in fluorescence activated cell sorting (FACS) buffer (PBS buffer containing 1% fetal bovine serum) for 1 hour. Cells were washed with FACS buffer and incubated with FITC-labeled goat anti-mouse IgG (H + L) secondary antibody (Life Technologies) on ice for 30 min. Then, all samples were washed using FACS buffer, followed by FACS analysis using a BD FACSCalibur system (BD Biosciences) [22].

### 2.5. Internalization Determination of PT

This assay was conducted as previously described [19, 23]. KYSE-450 cells were treated with the saturating concentration of PT at 4°C for 30 minutes. Unbound agent was removed by washing cells. Cells were then incubated at either 4°C or 37°C. At the indicated time points, the cell surface-bound PT was detected by flow cytometry with FITC-labeled anti-His-tag antibody.

### 2.6. *In Vitro* Cytotoxicity Assays

Esophageal cancer KYSE-450, KYSE-150, or KYSE-510 cells were plated in 96-well plates (5 × 10^3^ cells per well) and incubated with increasing concentrations of Pan or PT. Two days later, cell proliferation was determined using CCK-8 kit (Dojindo, Japan). The percentage of surviving cells was calculated using the following formula: [(A450 of experiment − A450 of background)/(A450 of untreated control − A450 of background)] × 100.

### 2.7. *In Vivo* Therapy Study

Esophageal cancer cells (1 × 10^7^ per mouse) were inoculated subcutaneously into the right flank of female BALB/c nude mice. When tumor volumes reached an average of about 150 mm^3^, the mice were randomly divided into 4 groups of 6 mice each. Mice were intravenously injected with control human IgG (10 mg/kg), Pan (10 mg/kg), PT (0.2 mg/kg), or PT (1 mg/kg) twice on indicated time point. Tumors were measured with digital calipers, and tumor volumes were calculated by the formula: volume = [length × (width)^2^]/2.

### 2.8. Immunoblotting

Cells were treated with indicated agents for 12 hours at 37°C. After washing, the cells were lysed in SDS lysis buffer and the cell lysates were subjected to SDS-PAGE and immunoblotted with antibodies against EGFR, p-EGFR (Tyr1068), p44/42 ERK1/2, p-ERK1/2 (Thr202/Tyr204), AKT, p-AKT (S473), Nrf2, Keap1, cleaved caspase-3, Bcl-2, Bcl-XL, or PARP.

### 2.9. Reactive Oxygen Species (ROS) Detection

The ROS detection assay was performed by using 2′,7′-dichlorofluorescin diacetate (DCFDA) staining (Beyotime Corporation). Briefly, DCFDA was diluted to 10 *μ*M with DMEM medium and added to the cells. After incubation for 1 hour, cells were exposed with different agent treatments at 37°C for 10 hours. Finally, fluorescence signal intensities indicating ROS levels were recorded by flow cytometry (Beckman Coulter) using excitation and emission spectra of 488/525 nm.

### 2.10. Apoptosis Analysis

Apoptosis analysis was performed by flow cytometry using established procedures [24]. For flow cytometry analysis, cells (5 × 10^6^/well) were plated in a 6-well plate and treated with Pan (10 nM) or PT (10 nM) for 16 h at 37°C. The cells were then labeled with annexin V and propidium iodide (PI) (Beijing Dingguo Biotechnology Co. Ltd, Beijing). Apoptotic rates were determined by FACSCalibur flow cytometer (BD Biosciences, Franklin Lakes, NJ) and analyzed by the FlowJo software. The percentage of the early apoptosis was calculated by annexin V(+) and PI(−), while the percentage of the late apoptosis was calculated by annexin V(+) and PI(+).

### 2.11. Statistical Analysis

Statistical analysis was performed by Student's unpaired *t*-test to identify significant differences unless otherwise indicated. Differences were considered significant at *p* < 0.05. Nonlinear regression analyses were used to fit curves. All statistic calculations were performed using the GraphPad Prism 5 software.

## 3. Results

### 3.1. Construction and Characterization of PT

**Figure 1:**
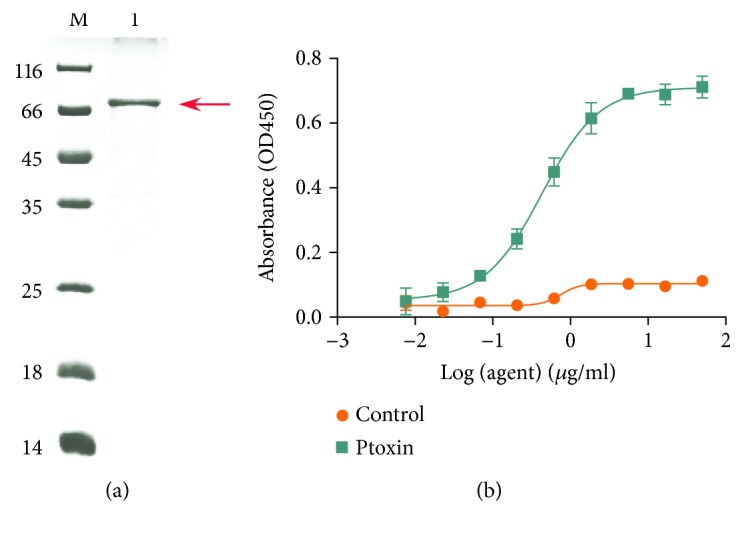
Characterization of Ptoxin. (a) SDS-PAGE analysis of Ptoxin. Lane 1: Ptoxin; M: protein marker. (b) ELISA analysis of Ptoxin-binding potency for recombinant EGFR antigen. EC50 for Ptoxin is 0.4304 *μ*g/mL (95% confidence interval (CI): 0.3571-0.5189 *μ*g/mL). IgG was used as control. Points: mean of 3 independent determinations; bars: SD.

In our previous study, we developed a novel antibody against EGFR, denoted as Pan [18]. To generate PT, the variable fragment of heavy chain and light chain from Pan was cloned into the pET-28a(+) plasmid containing PE38KEDL sequence, respectively. Then, the resulting plasmid was expressed in the soluble fraction of *Escherichia coli*. Finally, we purified the immunotoxin by nickel affinity chromatography and then verified the protein through SDS-PAGE (Figure 1(a)). Moreover, we also evaluated the binding activity of PT on antigen EGFR by ELISA assay. The result revealed that PT shows effective binding potency to recombinant EGFR (Figure 1(b)).

### 3.2. PT Is Effectively Internalized in Esophageal Carcinoma KYSE-450 and KYSE-150 Cells

**Figure 2:**
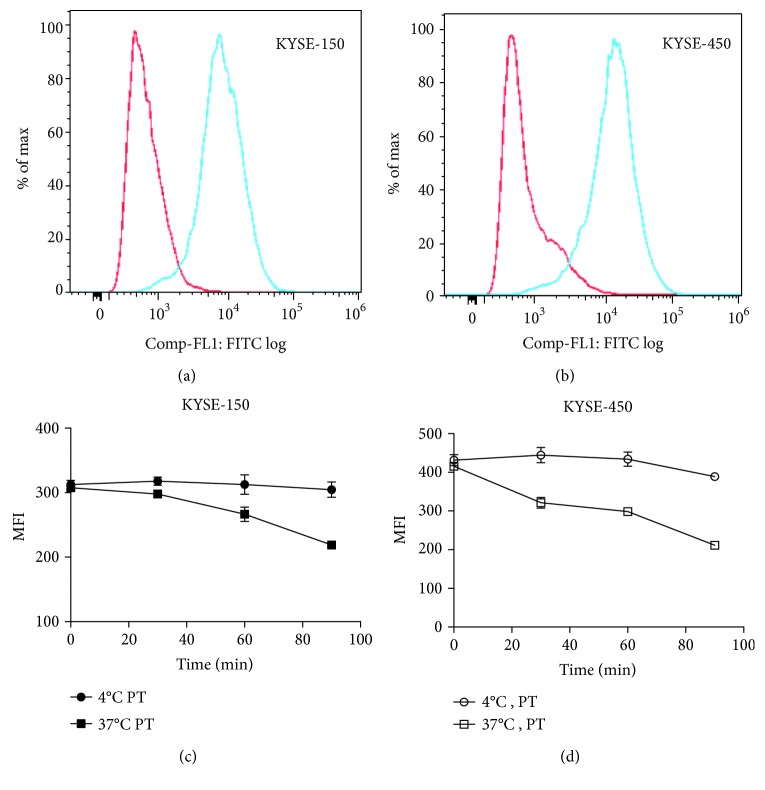
PT was rapidly internalized when bound to EGFR-overexpressed esophageal cancer cells. (a) Flow cytometric results, indicating cell surface EGFR expression in KYSE-150 cell lines. (b) Flow cytometric results, indicating cell surface EGFR expression in KYSE-450 cell lines. (c) Analysis of internalization rates of PT in KYSE-150 cells when binding to EGFR. (d) Analysis of internalization rates of PT in KYSE-450 cells when binding to EGFR. KYSE-150 or KYSE-450 cells were incubated with saturating level of PT (10 *μ*g/mL) for 30 minutes at 4°C. Unbound antibody conjugates were removed by washing cells. Cells were then incubated at either 4°C or 37°C. At the indicated time points, samples were detected by flow cytometry. Points: mean of 3 independent determinations; bars: SD.

First, we investigated the expression level of EGFR in esophageal carcinoma cells KYSE-450 and KYSE-150. As shown in Figures 2(a) and 2(b), both the two cells have strong EGFR-positive staining. Particularly, KYSE-450 has higher EGFR expression compared with KYSE-150 cells. As we know, efficient internalization of immunotoxin by target cells is necessary for delivering toxin (PE38) into cancer cells [25]. Therefore, the internalization of PT in KYSE-450 and KYSE-150 cells was evaluated. The surface level of PT markedly decreased on cells when shifted to 37°C over the course of a 90-minute study, suggesting rapid internalization of PT into KYSE-450 and KYSE-150 cells (Figures 2(c) and 2(d)).

### 3.3. PT Exhibits Superior Cytotoxicity in Esophageal KYSE-450 Cells

**Figure 3:**
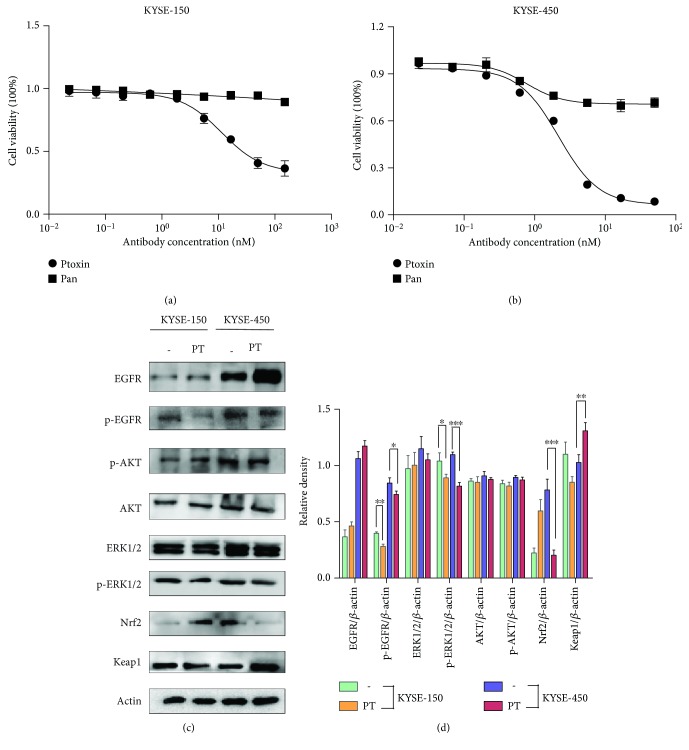
Treatment with PT inhibited the growth of esophageal cancer cells, and EGFR downstream signaling molecules were examined by western blot. (a) CCK-8 assay showing the inhibitory effects of Ptoxin on growth of KYSE-150 cells. IC50 is 11.43 nM (95% CI: 8.578-15.24 nM) for PT. Bars: SD. (b) CCK-8 assay showing the inhibitory effects of PT on growth of KYSE-450 cells. IC50 is 2.195 nM (95% CI: 1.904-2.532 nM) for PT. (c) Expression of key molecules involved in the EGFR-AKT, EGFR-ERK1/2, and Nrf2-Keap1 signaling pathway was tested in KYSE-150 and KYSE-450 cells upon medium alone (−) or PT (10 nM) treatment for 12 h. (d) Quantification of western blot signal intensity analysis is expressed relative to the *β*-actin loading control by using the ImageJ software. Data show the mean ± SD (3 independent experiments). ^∗^ *p* < 0.05, ^∗∗^ *p* < 0.01, and ^∗∗∗^ *p* < 0.001.

Next, we investigated the inhibitory effects of PT on the EGFR-expressing esophageal cancer cell lines. After 48 h incubation, PT inhibited the growth of KYSE-150 and KYSE-450 cancer cells in a dose-dependent manner, while Pan treatment only showed minimal inhibitory activity in the two cell lines (Figures 3(a) and 3(b)). And we also found that esophageal cancer cell line KYSE-510 with a low EGFR expression was hardly responsive to PT treatment (Figure S1). Notably, KYSE-450 cell line responded more sensitively to PT compared with KYSE-150 cell line. Next, we investigated the inhibitory effects of PT on the EGFR downstream signaling pathway in KYSE-450 and KYSE-150 cells.

Our results showed that PT treatment led to a significant decrease in the phosphorylation of EGFR and ERK1/2 in both KYSE-150 and KYSE-450 cells, which may partly explain antitumor effects of PT. As Nrf2 was a crucial downstream protein in the EGFR-Ras pathway, we investigated the expression of Nrf2 and Keap1, a repressor protein that binds to Nrf2 and modulates its degradation. Interestingly, we found that PT treatment caused a marked downregulation of Nrf2 protein expression in KYSE-450 cells, paralleled by an upregulation in Keap1 expression. In contrast, Nrf2 expression was upregulated and Keap1 was depressed in KYSE-150 cells (Figures 3(c) and 3(d)).

### 3.4. Enhancement of Reactive Oxygen Species (ROS) Accumulation and Apoptosis Induction May Explain the Superiority of PT in KYSE-450 Cells

**Figure 4:**
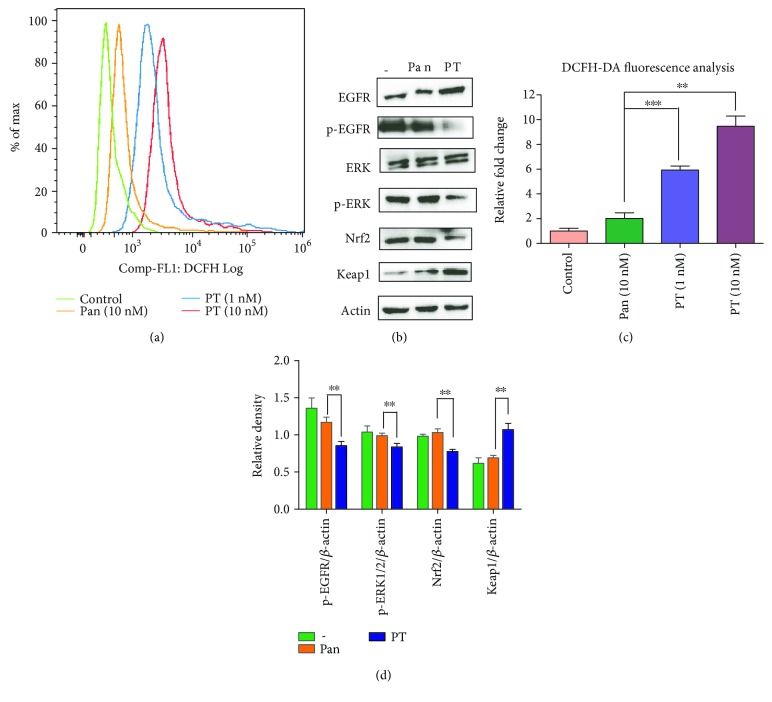
Treatment with PT resulted in ROS accumulation and caused repression of the Nrf2-Keap1 pathway. (a) KYSE-450 cells were treated with medium alone (control), Pan (10 nM), PT (1 nM), or PT (10 nM) for 10 hours, respectively, and flow cytometry was used to analyze the level of ROS accumulation in cells after DCFH-DA was added to stain the cells. (b) Key signaling molecules in response to treatments with Pan or PT treatment in KYSE-450 cells. Exponentially growing cells were treated with medium alone (−) or containing Pan (10 nM) or PT (10 nM) for 12 h before being analyzed. Then, EGFR, p-EGFR, ERK, p-ERK, Nrf2, and Keap1 were analyzed by western blot. (c) Bar graphic representations of the DCFH-DA fluorescence intensity upon different treatments relative to control. ^∗∗^ *p* < 0.01 and ^∗∗∗^ *p* < 0.001. (d) Quantification of western blot signal intensity analysis is expressed relative to the *β*-actin loading control by using the ImageJ software. Data show the mean ± SD (3 independent experiments). ^∗∗^ *p* < 0.01.

Nrf2 suppression always caused elevation of ROS [26–28]. Therefore, we examined the total ROS level when KYSE-450 cells were exposed to PT. As shown in Figures 4(a) and 4(c), we found that the treatment with PT either at a low dose (1 nM) or at a medium dose (10 nM) resulted in significant ROS release. We next assessed the expression of Nrf2 and Keap1 in KYSE-450 cells upon treatment with Pan or PT (Figures 4(b) and 4(d)). In consistent with previous result, PT significantly downregulated Nrf2 level and elevated Keap1 level in KYSE-450 cells compared with Pan treatment. Moreover, phosphorylation of EGFR and ERK1/2 was also regressed in PT-treated cells.

**Figure 5:**
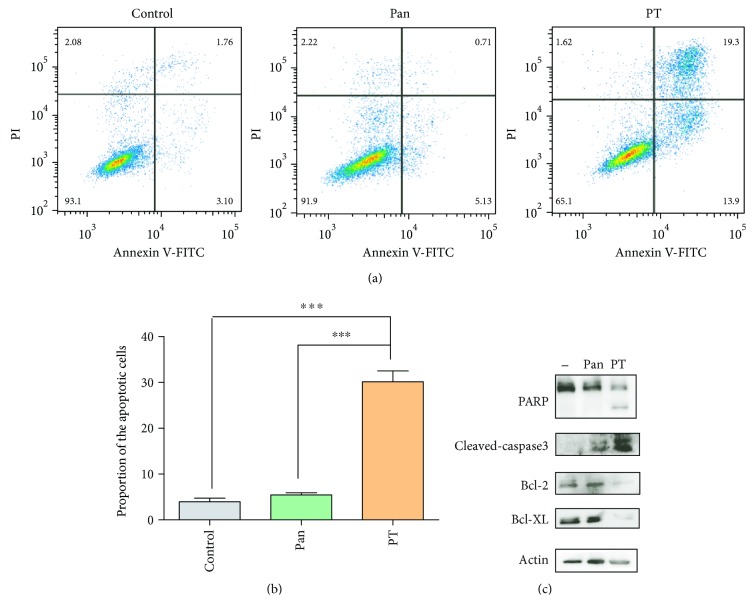
PT treatment resulted in caspase-3-dependent apoptosis in KYSE-450 cells. (a) Induction of apoptosis in KYSE-450 cells with medium alone (control), PT (10 nM), or Pan (10 nM) treatment for 16 h. Apoptosis proportion was measured by flow cytometry. (b) Statistical analysis of the percentage of the apoptotic cells. Data was shown with mean ± SD. ^∗∗∗^ *p* < 0.001. (c) Apoptosis-related protein (PARP, cleaved caspase-3, BcL-XL, or Bcl-2) was examined in KYSE-450 cells when treated with medium alone (−), Pan (10 nM), or PT (10 nM) for 16 h.

As we know, ROS plays an important role in cancer therapy by activating apoptosis [29, 30]. We further investigated if PT treatment indeed induced apoptosis by FACS and western blot. As shown in Figures 5(a) and 5(b), the percentage of apoptotic cells was significantly increased in the PT-treated cells compared to Pan-treated cells. To further reveal the effects of PT on apoptosis, we measured the expression levels of a set of apoptosis-relevant proteins. Results in Figure 5(c) showed that PT treatment resulted in marked PARP and caspase-3 cleavage, which were classic apoptotic initiators. In consistent with these results, downregulation of antiapoptotic proteins including Bcl-2 and Bcl-XL was also observed upon PT treatment. Taken together, these data demonstrated that PT may exert its antitumor activity through causing ROS accumulation and inducing apoptosis in KYSE-450 cells.

### 3.5. PT Exhibits Potent Antitumor Activity and Good Tolerance *In Vivo*

**Figure 6:**
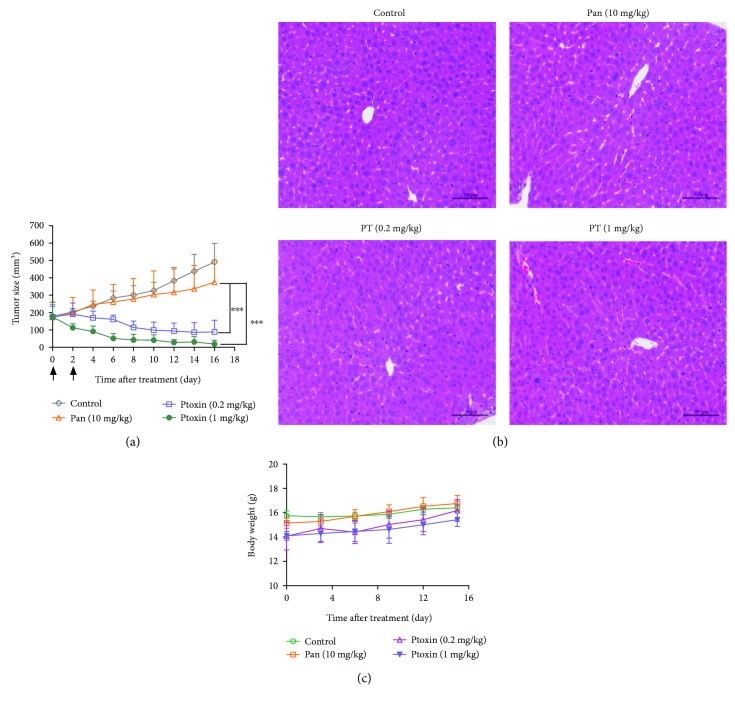
*In vivo* efficacy of PT in the KYSE-450 xenograft tumor model. (a) Mean tumor volumes of mice xenografted with KYSE-450 cells and treated with PT. There were 6 mice per treatment group. PT treatment started as indicated in the graphs (black arrows). Error bars show ±SD (^∗∗∗^ *p* < 0.001). (b) Histological examination was conducted in nude mice post 2 weeks after the last injection with Pan or PT. Representative images (magnification, ×400) of the liver from nude mice injected with indicated agents for two times were obtained by staining with hematoxylin and eosin. Scale bars: 100 *μ*m. (c) Effect of PT on nude mice body weight was determined using KYSE-450 tumor-bearing nude mice. Mice were weighed at regular intervals during the whole period to monitor therapy-related toxicity.

To further evaluate the inhibitory activity of PT, we investigated the therapeutic effect of PT in nude mice bearing established KYSE-450 and KYSE-150 cancer cells. As shown in Figure 6(a), the treatment with PT (0.2 mg/kg) resulted in marked tumor growth inhibition and treatment with 1 mg/kg PT caused nearly complete tumor regression on KYSE-450 tumor xenografts, whereas only marginal tumor growth inhibition was observed in tumor-bearing mice treated with Pan (10 mg/kg). In consistent with *in vitro* results, PT treatment at a dose of 1 mg/kg only shows limited antitumor potency on KYSE-150 tumor xenografts (Figure S2).

To assess the therapy-related unspecific toxicity, organ toxicity and body weight were monitored in nude mice bearing established subcutaneous KYSE-450 tumor xenografts. As shown in Figure 6(b), hematoxylin and eosin (H&E) staining showed that no marked liver toxicity was observed in PT-treated mice, even at high dose (1 mg/kg). Moreover, the average body weight change of PT injection group in treatment period has no significant difference compared to that of control IgG or Pan treatment group (Figure 6(c)).

## 4. Discussion

EGFR, an extensively studied membrane-bound receptor tyrosine kinase (RTK), is commonly overexpressed in esophageal cancers [31]. However, current established EGFR inhibitors such as mAbs have limited anticancer efficacy in EGFR-positive esophageal cancers, which create the need for better therapeutic avenue [32, 33]. Considering the presence of Fc region, the large size of traditional mAbs showed poor tumor penetration and decreased access to and killing of interior tumor cells [34]. Therefore, development of immunotoxin with smaller size and more potent activity has become a powerful approach to overcome traditional anti-EGFR antibody limitations.

In our study, we developed and characterized an EGFR-targeted immunotoxin (denoted as PT) derived from Pan, which was previously reported by our team [18]. To observe the inhibitory effects of PT *in vitro*, we utilized 3 esophageal cancer cell lines including KYSE-450, KYSE-150, and KYSE-510. PT shows potent inhibitory potency on KYSE-450 cells and moderate inhibitory effects on KYSE-150 cells. However, KYSE-510 was hardly responsive to PT treatment. We further found that PT more effectively inhibits the growth of KYSE-450 and KYSE-150 cells than Pan. Particularly, PT shows potently inhibitory effects on KYSE-450 cells. We further explored the mechanism of action of PT. Data indicated that the phosphorylation of EGFR and ERK1/2 was inhibited in both KYSE-450 and KYSE-150 cells when treated with PT. Recently, the Nrf2-Keap1 pathway is always associated with redox homeostasis and cancer therapy [26, 35, 36]. Many studies indicated that Nrf2 expression increased cancer chemoresistance and enhanced tumor growth and proliferation [16, 17]. Notably, we found that Nrf2 level was significantly regressed and Keap1 level was markedly elevated in PT-treated KYSE-450 cells, which reveals that the Nrf2 antioxidant response pathway was inhibited and may crosstalk with the EGFR-ERK1/2 pathway in KYSE-450 cells. However, the exact mechanism of this intricate connectivity between the Nrf2-Keap1 and EGFR-ERK1/2 pathways remains to be explored.

Clearly, significant elevation of ROS accumulation was verified in PT-treated KYSE-450 cells compared to Pan-treated cells. Moreover, oxidative stress-dependent apoptosis was also found when treated with PT with a longer period. Therefore, we speculated PT treatment resulted in inhibition of the Nrf2-Keap1 antioxidant pathway and then effectively increased ROS accumulation and induced apoptosis of KYSE-450 cells, which may also partly explain a stronger inhibitory effect of PT treatment on KYSE-450 cells than on KYSE-150 cells. Recently, we verified the effects on additional esophageal cancer cell line EC9706. Results revealed that PT treatment also induced ROS production and caused inhibition of the Nrf2-Keap1 pathway on EC9706 cell line (Figure S3). Further studies will be performed to explore if the effects existed in other esophageal cancer cell lines.

Consistent with these results above, we hypothesized that PT treatment may firstly inhibit the activation of the EGFR-ERK1/2 signaling pathway, and then resulted in suppression of the Nrf2-Keap1 antioxidant pathway, which was a potential mechanism underlying ROS release. Finally, ROS accumulated and oxidative stress-dependent apoptosis happened, which may directly explain the strong inhibitory effects of PT in KYSE-450 cells.

Overall, our study is the first to demonstrate that ROS accumulation by suppression of the Nrf2-dependent antioxidant response, which results in alterations in redox homeostasis, is a new mechanism underlying the potent antitumor activity of EGFR-targeted immunotoxin. As the cytotoxic mechanism of PT is markedly distinct from other EGFR-targeted agents, it may be a promising approach for EGFR^+^ esophageal cancer patients in the future.

## 5. Conclusions

Taken together, our results revealed that PT was effective in suppressing the growth of KYSE-150 and KYSE-450 cells, especially for KYSE-450 cells. We for the first time indicated that PT may exert antitumor activity on KYSE-450 cells through promoting ROS production and inducing apoptosis via inhibition of the Nrf2-Keap1 pathway. Given these, PT has a great potential to be a promising therapeutic candidate for treating EGFR^+^ esophageal cancers.

## Data Availability

The data used to support the findings of this study are included within the article.

## Supplementary Materials

Supplementary Materials Figure S1: the inhibitory effect of PT on the growth of KYSE-510 esophageal cancer cell line that has a low EGFR expression was examined utilizing CCK-8 assay. Figure S2: *in vivo* antitumor activity of PT was examined in the KYSE-150 xenograft tumor model. Figure S3: PT treatment induced ROS accumulation and inhibited the Nrf2 pathway on esophageal cancer EC9706 cell line.
