# Supplementary material for: EGFR-Targeted Immunotoxin Exerts Antitumor Effects on Esophageal Cancers by Increasing ROS Accumulation and Inducing Apoptosis via Inhibition of the Nrf2-Keap1 Pathway
Source: J Immunol Res. 2018 Nov 25;2018:1090287. doi: 10.1155/2018/1090287 (PMC6286775; doi:10.1155/2018/1090287)
Supplement: Supplementary Materials — Figure S1: the inhibitory effect of PT on the growth of KYSE-510 esophageal cancer cell line that has a low EGFR expression was examined utilizing CCK-8 assay. Figure S2: in vivo antitumor activity of PT was examined in the KYSE-150 xenograft tumor model. Figure S3: PT treatment induced ROS accumulation and inhibited the Nrf2 pathway on esophageal cancer EC9706 cell line. [file 1090287.f1.pdf]

Supplementary Materials

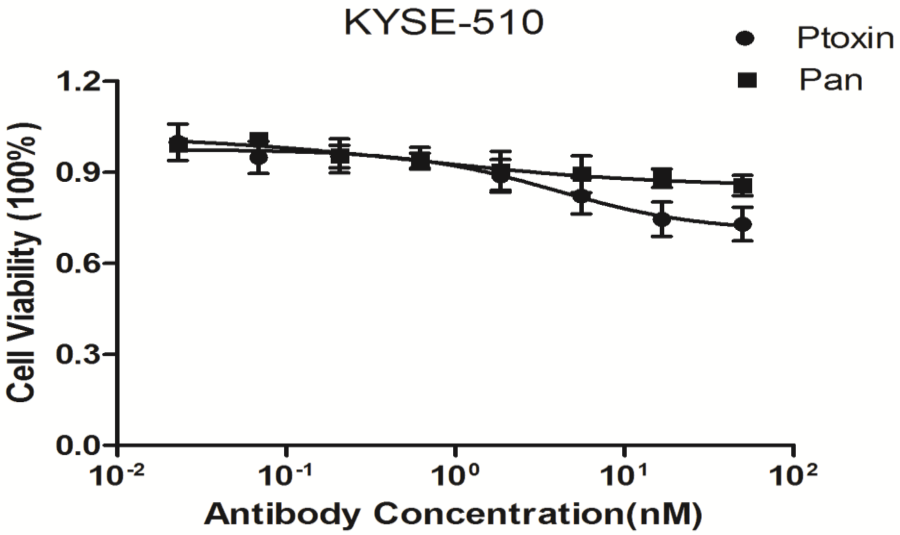

FIGURE S1: The inhibitory effect of PT on the growth of KYSE-510 esophageal cancer cell line that has a low EGFR expression was examined by CCK-8 assay.

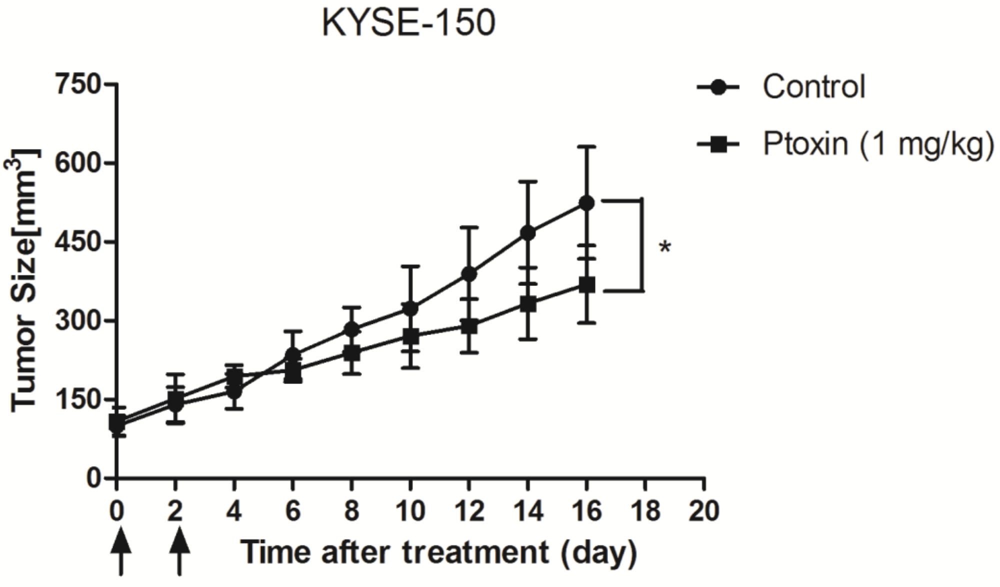

FIGURE S2: *In vivo* anti-tumor activity of PT was examined in the KYSE-150 xenograft tumor model. Mean tumor volumes of mice xenografted with KYSE-150 cells and treated with PT. There were 6 mice per treatment group. PT treatment started as indicated in the graphs (black arrows). Error bars show ± SD. (*p < 0.05).

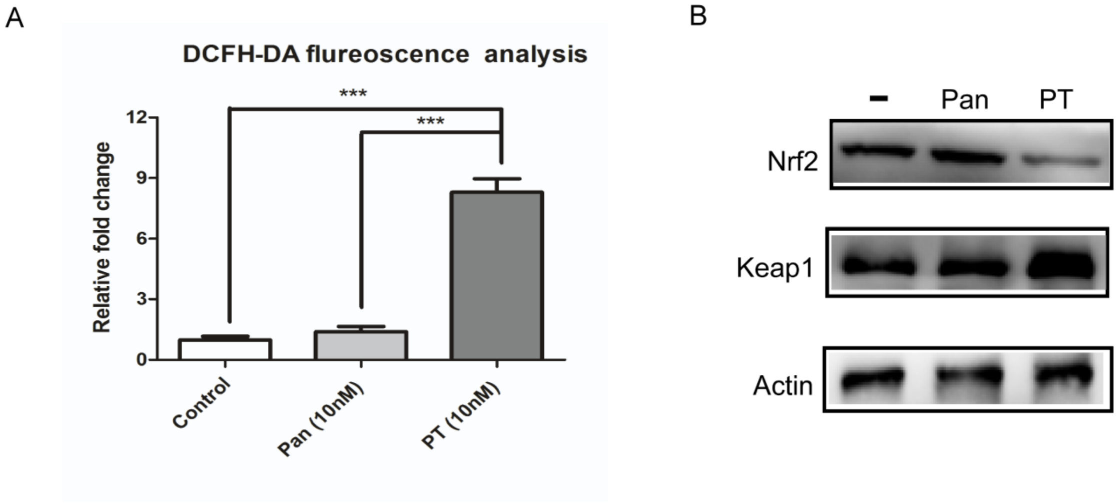

FIGURE S3: PT treatment induced ROS accumulation and inhibited Nrf2 pathway in esophageal cancer EC9706 cell line. (a) EC9706 cells were treated with medium alone (control), Pan (10 nM) or PT (10 nM) for 12 hours respectively, and flow cytometry was used to analyze the level of ROS accumulation in cells after DCFH-DA was added to stain the cells. Bar graphic representations of the DCFH-DA fluorescence intensity upon different treatments relative to control. ***p < 0.001. (b) Nrf2 signaling pathway was inhibited in EC9706 cells upon treatment with PT. Exponentially growing cells were treated with medium alone (-) or containing Pan (10 nM) or PT (10 nM) for 12 h before being analyzed.

Then the level of Nrf2 and Keap1 were analyzed by western blot. β-actin was used as loading control.
